# Vancomycin-Induced Drug Reaction with Eosinophilia and Systemic Symptoms (DRESS) Syndrome Masquerading as Elusive Sepsis

**DOI:** 10.1155/2019/1625010

**Published:** 2019-04-10

**Authors:** Sumon Roy, Vinay P. Goswamy, Kirolos N. Barssoum, Devesh Rai

**Affiliations:** Department of Medicine, Rochester General Hospital, Rochester Regional Health System, Rochester, NY 14621, USA

## Abstract

We present a unique case of vancomycin-induced drug reaction with eosinophilia and systemic symptoms (DRESS) syndrome masquerading as elusive endocarditis. A 37-year-old female actively using intravenous drugs presented with worsening right upper extremity pain, fever, and chills. Workup revealed methicillin-resistant staphylococcus aureus (MRSA) bacteremia and multiple right-sided septic pulmonary emboli. Echocardiogram was negative for vegetation. Vancomycin was initiated for bacteremia management suspected secondary to right upper extremity abscesses. However, despite resolution of abscesses, fevers persisted, raising suspicion for endocarditis not detected by echocardiogram. On hospital day 25, the patient began showing signs of DRESS syndrome, ultimately manifesting as transaminitis, eosinophilia, and a diffuse, maculopapular rash. Vancomycin was switched to Linezolid and she improved on high dose steroids. The persistent fevers throughout this hospital course were thought to be an elusive endocarditis before DRESS syndrome fully manifested. Although Vancomycin-induced DRESS is uncommon, this case highlights the importance of identifying early signs of significant adverse effects.

## 1. Introduction

Drug reaction with eosinophilia and systemic symptoms (DRESS) syndrome is seldom seen, distinct, and a potentially life-threatening drug-induced Type IV hypersensitivity reaction that is frequently associated with reactivation of latent HHV-6 infections [1]. Symptoms characteristically start between two and six weeks after starting the offending drug and include a morbilliform cutaneous rash involving greater than 50% of body surface area, fevers, lymphadenopathy, and frequently multiorgan involvement. Laboratory findings generally include leukocytosis with marked eosinophilia, increased serum alanine aminotransferase, and atypical lymphocytosis. The pathogenesis includes an expansion of activated CD4 and CD8 cells which is thought to contribute to a reactivation of herpesvirus infections in most, although not all, cases. Although the incidence is unknown, one prospective study estimated an annual incidence of 0.9/100,000 [2]. The most commonly implicated agents include allopurinol and the anticonvulsive medications lamotrigine, carbamazepine, and phenytoin. Sulfonamides such as vancomycin are rarely associated with DRESS syndrome. In this report, we present a unique case of vancomycin-induced DRESS syndrome manifesting as an elusive endocarditis for several weeks before ultimately developing into florid eosinophilia and systemic symptoms.

## 2. Case Report

A 37-year-old female with a medical history significant for intravenous drug abuse initially presented to the Emergency Department (ED) complaining of right upper extremity pain and swelling of over the past day. Suspecting superficial thrombophlebitis, she was discharged from the ED with a prescription for clindamycin. However, the patient subsequently returned to the ED two days later with worsening right upper extremity pain and swelling now associated with fever and chills.

Vital signs on admission were notable for temperature 38.1°C, blood pressure 152/90 mmHg, and heart rate 124 beats per minute. Physical exam revealed the right forearm to be significantly swollen on the medial aspect, with the area notably erythematous and warm to touch. Laboratory data showed a leukocytosis of 14,300/*μ*l predominantly neutrophilic. Chest X-ray showed bilateral airspace disease, and subsequent computed tomography (CT) chest revealed innumerable right pulmonary septic emboli. Transthoracic echocardiogram and transesophageal echocardiogram were negative for vegetation. Broad spectrum antibiotics were initiated pending blood culture data, which resulted by the second day as positive for methicillin resistant staphylococcus aureus (MRSA) bacteremia in 4 out of 4 bottles. The patient was then transitioned to vancomycin monotherapy for an extended time course.

Surveillance cultures done on the fourth day of hospitalization were negative. In the interval, the patient underwent multiple incision and drainage procedures of several abscesses on her right upper extremity, the largest of which measured 3 cm in diameter.

Despite appropriate antibiotic therapy, the patient was spiking intermittent fevers. Investigation with repeat CT scan of the chest revealed bilateral loculated empyema. The patient subsequently underwent bronchoscopy and eventually right video-assisted thoracic surgery (VATS) procedure that was converted to open left thoracotomy for evacuation of loculated empyema, decortication, and placement of chest tube. Pleural fluid cultures were positive for MRSA.

The patient remained persistently febrile, with workup not revealing an identifiable cause. Surveillance blood cultures remained negative. Repeat CT scan of the chest revealed new small filling defect in the left lower lobe segmental pulmonary artery; however, the right sided filling defects had resolved. CT scan of the abdomen was pursued searching for other causes of fever but was unremarkable.

**Figure 1:**
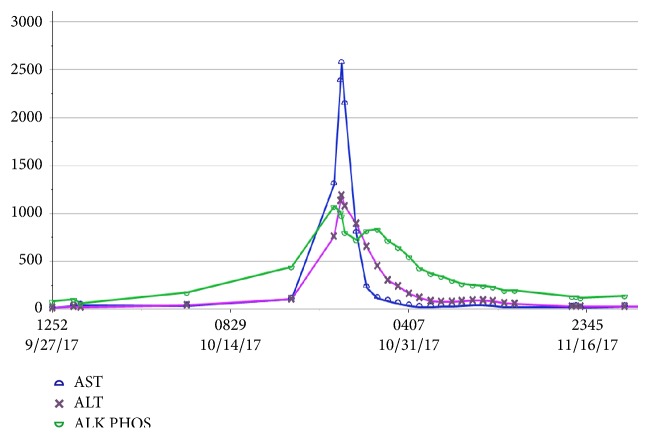
Graphical representation of laboratory value trends of aspartate aminotransferase (AST), alanine aminotransferase (ALT), and alkaline phosphatase (Alk Phos) over the course of the admission. Each marker is noted to peak approximately 3-4 weeks into admission.

**Figure 2:**
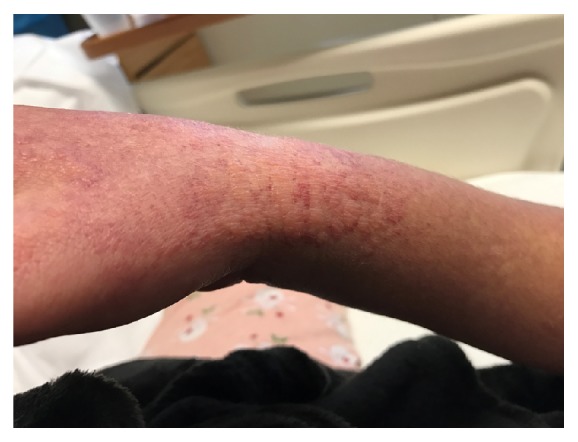
Representative photograph of the maculopapular rash noted on right upper extremity is shown in the image above. Similar presentation was also noted on left upper extremity (not shown).

**Figure 3:**
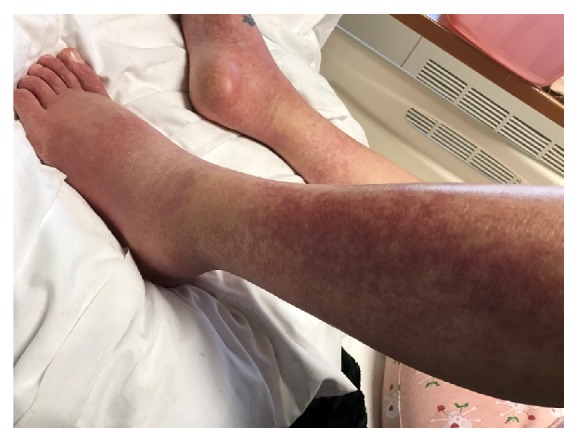
Representative photograph of maculopapular rash noted on left lower extremity is shown above. Similar presentation was also noted on right lower extremity (in background).

**Figure 4:**
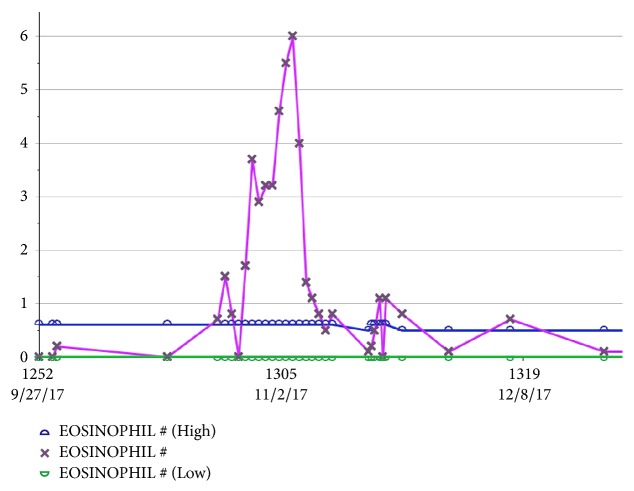
Graphical representation of the absolute eosinophil count (Eosinophil #) (pink line) over the course of the admission, with the normal range demarcated by Eosinophil # (Low) (green line) as the minimum and the Eosinophil # (High) (blue line) as the maximum values and blue (max) and green (min) values. Absolute eosinophil count increased several fold above the upper limit of normal approximately 3-4 weeks into admission.

On day 22 of vancomycin therapy, liver enzymes were noted to be uptrending, with aspartate aminotransferase peaking at 2,563 units/L, alanine aminotransferase peaking at 1,192 units/L, and alkaline phosphatase peaking at 1,076 units/L (Figure 1). Within two days, new leukocytosis was noted and continued to uptrend for the next few days. On day 25 of vancomycin therapy, a diffuse maculopapular rash erupted involving bilateral upper and lower extremities as well as the upper chest (Figures 2 and 3). At this time, suspecting vancomycin-induced DRESS syndrome, antibiotic therapy was switched to ceftaroline. By day 29, the eosinophil count began uptrending, with absolute eosinophil count noted at 600/*μ*l on day 35 (Figure 4). She concurrently developed acute kidney injury with blood urea nitrogen peaking at 27 mg/dL and serum creatinine peaking at 1.4 mg/dL (baseline creatinine 0.6 mg/dL). Multiorgan system involvement was noted as hepatic and renal dysfunction was evident on laboratory workup, and the patient subsequently developed cardiopulmonary instability requiring management in the medical intensive care unit. The patient eventually improved on glucocorticoid management that was planned for an extended taper over 12 weeks. The antibiotic was changed to Linezolid to finish six-week course of antibiotic for MRSA bacteremia.

## 3. Discussion

Due to the variability of the presentation of DRESS syndrome, diagnosis is largely based on clinical suspicion in the setting of a newly administered medication in the past two to six weeks. The European Registry of Severe Cutaneous Adverse Reactions (regiSCAR) has developed a scoring system which may aid clinicians to order certain laboratory tests but mainly serves to confirm cases retrospectively. Early consultations to dermatology can also be valuable. Histopathology via skin biopsy which reveals perivascular lymphocytic infiltrate with the presence of eosinophils is consistent with DRESS syndrome.

Vancomycin itself has been reported as a trigger of DRESS syndrome in a limited number of case series and case reports. In a 2016 review of hypersensitivity reactions reported from 1982 to 2015 associated with Vancomycin, Minhas et al. identified 71 total hypersensitivity reactions of which 16 were due to DRESS syndrome [3]. Unfortunately, regiSCAR scoring methods were only reported in four of these cases.

In a three-year retrospective chart review of 32 cases of DRESS syndrome at LA County and Keck Medical Centers by Lam et al., 12 cases surprising were found to be triggered by vancomycin. All cases of DRESS in this series were diagnosed with the regiSCAR scoring system.

Dress syndrome is a severe drug reaction that involves the skin as well as organs. Differential diagnoses could include Stevens-Johnson syndrome (SJS), toxic epidermal necrolysis (TEN), and acute generalized exanthematous pustulosis (AGEP), all of which are considered severe cutaneous adverse reactions (SCARs) to medications [4].

SJS and TEN often show early manifestations within days but can also have delayed presentation at approximately four weeks, similar to DRESS syndrome. Cutaneous reactions, which are dark, macular, irregularly shaped lesions on the trunk and face, can be preceded by a mild, nonspecific prodrome. Within days, the lesions progress to flaccid bullae due to dermal- epidermal detachment, which tend to necrotize exposing the dermis [5]. The extent of cutaneous involvement of this disease spectrum ranges from STS (<10% body surface area) and STS/TEN overlap (10-30% body surface area) to TEN (>30% body surface area) [5].

AGEP typically manifests sooner, often within 48 hours, after administration of various medications including sulfonamides, ketoconazole, fluconazole, terbinafine, and diltiazem. The rash typically consists of innumerous pustules with minimal mucous membrane involvement. Neutrophilia is prominent on blood work [6].

Hypereosinophilic syndromes can also present with cutaneous manifestations that have a wide array of lesions. The most frequently reported reactions in this context include pruritic maculo-papules, urticarial manifestations, and angioedema [7].

Drug reactions secondary to vancomycin span a wide spectrum. The most common reaction is Red Man syndrome, manifesting as facial flushing, pruritis, and an erythematous rash of the upper body. The presentation often resembles a hypersensitivity reaction, though the true mechanism remains unclear [8–10]. Treatment generally consists of decreasing the rate of infusion and administering antihistamine agents [11].

Dermatological hypersensitivity reactions may range from a simple skin rash to Linear IgA Bullous Dermatosis (LABD), in which IgA autoantibody formation can be triggered by vancomycin [12]. Other dermatologic conditions include exfoliative dermatitis, leukocytoclastic vasculitis, SJS, and TEN. In the more dangerous clinical contexts, vancomycin must be stopped.

Vancomycin has caused various other drug reactions to include nephrotoxicity, hematologic abnormalities such as immune thrombocytopenia, and drug fever [3, 13]. Anaphylaxis, although rare, has also been associated with vancomycin administration [3].

Two cases of DRESS have been associated with a cross reactivity between vancomycin and teicoplanin, although in the latter the reaction to teicoplanin appears to be doubtful as symptoms started mere three days after initiation of the drug [14, 15].

The case presented offers multiple unique perspectives. The patient initially presented with fever and chills and was found to have multiple septic emboli to lung along with bilateral loculated empyema. The hospital course appeared to resemble that of sepsis for which the source remained elusive, though the patient was managed for thrombophlebitis, loculated empyema, and septic emboli. Additionally, the rash manifested four days prior to the evolution of eosinophilia, liver dysfunction, and acute kidney injury. This sequence of events represents an atypical presentation of DRESS syndrome.

Once DRESS syndrome is suspected, a prompt withdrawal of any high-risk medications serves as the initial and most important step in management. Supportive measures such as fluids, nutrition, and electrolyte balance are also important. Both high dose topical and systemic corticosteroid treatment have been described although no randomized trials exist to support their use. The decision to use topical or systemic steroids is usually based on the severity of the reaction. In most of the above described cases, systemic corticosteroids were used with success. Treatment with cyclosporine has only been described successfully in a few cases, although this remains a second line treatment to corticosteroids [16, 17].

## References

[1] Descamps V., Valance A., Edlinger C. (2001). Association of human herpesvirus 6 infection with drug reaction with eosinophilia and systemic symptoms. *JAMA Dermatology*.

[2] Muller P., Dubreil P., Mahe A. (2003). Drug Hypersensitivity Syndrome in a West-Indian population. *European Journal of Dermatology*.

[3] Minhas J. S., Wickner P. G., Long A. A., Banerji A., Blumenthal K. G. (2016). Immune-mediated reactions to vancomycin: A systematic case review and analysis. *Annals of Allergy, Asthma & Immunology*.

[4] Behera S. K., Das S., Xavier A. S., Selvarajan S. (2018). DRESS syndrome: a detailed insight. *Hospital Practice*.

[5] Dodiuk-Gad R. P., Chung W.-H., Valeyrie-Allanore L., Shear N. H. (2015). Stevens–Johnson syndrome and toxic epidermal necrolysis: An update. *American Journal of Clinical Dermatology*.

[6] Szatkowski J., Schwartz R. A. (2015). Acute generalized exanthematous pustulosis (AGEP): a review and update. *Journal of the American Academy of Dermatology*.

[7] Plötz S. G., Hüttig B., Aigner B. (2012). Clinical overview of cutaneous features in hypereosinophilic syndrome. *Current Allergy and Asthma Reports*.

[8] Healy D. P., Sahai J. V., Fuller S. H., Polk R. E. (1990). Vancomycin-induced histamine release and “red man syndrome”: comparison of 1- and 2-hour infusions. *Antimicrobial Agents and Chemotherapy*.

[9] Newfield P., Roizen M. F. (1979). Hazards of rapid administration of vancomycin. *Annals of Internal Medicine*.

[10] Polk R. E., Healy D. P., Schwartz L. B. (1988). Vancomycin and the red-man syndrome: Pharmacodynamics of histamine release. *The Journal of Infectious Diseases*.

[11] Renz C. L., Thurn J. D., Finn H. A., Lynch J. P., Moss J. (1998). Oral Antihistamines Reduce the Side Effects from Rapid Vancomycin Infusion. *Anesthesia & Analgesia*.

[12] Lammer J., Hein R., Roenneberg S., Biedermann T., Volz T. (2019). Drug-induced linear IgA bullous dermatosis: a case report and review of the literature. *Acta Dermato-Venereologica*.

[13] Gerstein W., Colombo E., Harji F. (2018). Documented vancomycin-induced severe immune-mediated thrombocytopaenia. *BMJ Case Reports*.

[14] Tamagawa-Mineoka R., Katoh N., Nara T., Nishimura Y., Yamamoto S., Kishimoto S. (2007). DRESS syndrome caused by teicoplanin and vancomycin, associated with reactivation of human herpesvirus-6. *International Journal of Dermatology*.

[15] Kwon H.-S., Chang Y.-S., Jeong Y.-Y. (2006). A case of hypersensitivity syndrome to both vancomycin and teicoplanin. *Journal of Korean Medical Science*.

[16] Kirchhof M. G., Wong A., Dutz J. P. (2016). Cyclosporine treatment of drug-induced hypersensitivity syndrome. *JAMA Dermatology*.

[17] Zuliani E., Zwahlen H., Gilliet F., Marone C. (2005). Vancomycin-induced hypersensitivity reaction with acute renal failure: Resolution following cyclosporine treatment. *Clinical Nephrology*.

